# Accelerating Kenya’s progress to 2030: understanding the determinants of under-five mortality from 1990 to 2015

**DOI:** 10.1136/bmjgh-2017-000655

**Published:** 2018-05-24

**Authors:** Emily C Keats, William Macharia, Neha S Singh, Nadia Akseer, Nirmala Ravishankar, Anthony K Ngugi, Arjumand Rizvi, Emma Nelima Khaemba, John Tole, Zulfiqar A Bhutta

**Affiliations:** 1 Centre for Global Child Health, The Hospital for Sick Children, Toronto, Ontario, Canada; 2 Faculty of Health Sciences, Aga Khan University, Nairobi, Kenya; 3 Centre for Maternal, Adolescent, Reproductive, and Child Health, London School of Hygiene & Tropical Medicine, London, UK; 4 Dalla Lana School of Public Health, University of Toronto, Toronto, Canada; 5 Independent consultant, Nairobi, Kenya; 6 Division of Woman and Child Health, Aga Khan University, Karachi, Pakistan; 7 African Population and Health Research Center, Nairobi, Kenya

**Keywords:** child health, health policy, health systems, nutrition, maternal health

## Abstract

**Introduction:**

Despite recent gains, Kenya did not achieve its Millennium Development Goal (MDG) target for reducing under-five mortality. To accelerate progress to 2030, we must understand what impacted mortality throughout the MDG period.

**Methods:**

Trends in the under-five mortality rate (U5MR) were analysed using data from nationally representative Demographic and Health Surveys (1989–2014). Comprehensive, mixed-methods analyses of health policies and systems, workforce and health financing were conducted using relevant surveys, government documents and key informant interviews with country experts. A hierarchical multivariable linear regression analysis was undertaken to better understand the proximal determinants of change in U5MR over the MDG period.

**Results:**

U5MR declined by 50% from 1993 to 2014. However, mortality increased between 1990 and 2000, following the introduction of facility user fees and declining coverage of essential interventions. The MDGs, together with Kenya’s political changes in 2003, ushered in a new era of policymaking with a strong focus on children under 5 years of age. External aid for child health quadrupled from 40 million in 2002 to 180 million in 2012, contributing to the dramatic improvement in U5MR throughout the latter half of the MDG period. Our multivariable analysis explained 44% of the decline in U5MR from 2003 to 2014, highlighting maternal literacy, household wealth, sexual and reproductive health and maternal and infant nutrition as important contributing factors. Children living in Nairobi had higher odds of child mortality relative to children living in other regions of Kenya.

**Conclusions:**

To attain the Sustainable Development Goal targets for child health, Kenya must uphold its current momentum. For equitable access to health services, user fees must not be reintroduced in public facilities. Support for maternal nutrition and reproductive health should be prioritised, and Kenya should acknowledge its changing demographics in order to effectively manage the escalating burden of poor health among the urban poor.

**Key questions**

**What is already known?**

- Earlier studies in Kenya have reported a range of factors proximal to the child that influence under-five mortality, though the impact of broader health system determinants have been less well characterised.

**What are the new findings?**

- Macro-level factors, including the introduction of health facility user fees, the proliferation of child health-specific policies and bolstered external aid for child health helped to shape under-five mortality trends from 1990 to 2015.
- Improved maternal sexual and reproductive health indicators contributed substantially to the decline in child mortality from 2003 to 2014.
- Children living in Nairobi had higher odds of child mortality relative to children living in other regions of Kenya.

**What do the new findings imply?**

- Along with strengthening the health workforce in Kenya’s devolved nation, reproductive health and the health of urban poor populations should be a focus of both national-level and county-level governments moving forward.

## Background

Kenya today is a fast-growing and pivotal East African economy and a fledgling democratic state. While major social and political advancements have taken place, healthcare across Kenya has not kept pace. Kenya fell short of its Millennium Development Goal (MDG) targets for maternal and child mortality,^1 2^ showing inconsistent progress in trends between 1990 and 2015. Despite recent improvements, estimates suggested an increase in the under-five mortality rate (U5MR) between 1990 and 2000.^3^ Currently, the U5MR is 51.3/1000 and is poised to reach the 2030 Sustainable Development Goal (SDG) target of 25 deaths/1000 live births at the current annual rate of reduction.^3^ Maternal and neonatal mortality reduction will require accelerated efforts to reach the SDG targets of 70 maternal deaths/100 000 live births and 12 neonatal deaths/1000 live births, respectively.^3^

In any country, progress in health may be partially attributed to the successful implementation of individual interventions, but outcomes, to a large extent, are determined by health system functioning. The building blocks of a health system—governance and legislation, health financing, workforce, infrastructure and commodities, service delivery and health information systems—form the most distal inputs on a pathway to achieving impact (online supplementary eFigure 1). Limitations in any one of these core areas can lead to poor service delivery, low and inequitable intervention coverage and negative health outcomes for mothers and children. Recently, Countdown to 2015-led efforts have been made to better understand how to assess health system inputs in a standardised manner.^4^ However, to date, there is a paucity of robust and systematically collected information from Kenya on health systems performance, financial allocations for reproductive, maternal, newborn, child and adolescent health (RMNCAH) and other contextual factors that could have affected progress throughout the MDG period. Understanding these inputs and their impact will help to inform priorities and strategies for action leading up to 2030.

10.1136/bmjgh-2017-000655.supp1 Supplementary file 1

Following our recent analysis of intervention coverage, equity and mortality (maternal, newborn and child) trends in Kenya throughout the MDG period (1990–2015),^3^ we subsequently aimed to empirically evaluate the drivers of change in under-five child mortality, taking into account the complex country context and underlying determinants. Child health was a focus of the current quantitative analysis due to data limitations with maternal and neonatal mortality. To understand the various determinants, we (1) undertook a standardised analysis of RMNCAH systems, policies, workforce and financing in Kenya over the MDG period, using the Kenya Countdown case study conceptual framework as a reference (online supplementary eFigure 1) and (2) examined proximal household and individual-level factors, along with intermediary factors, that may have contributed to change in U5MR.

## Methods

For both the main case study^3^ and the current analysis, we used a Countdown-adapted conceptual framework (online supplementary eFigure 1) to guide our approach to understanding RMNCAH in Kenya. This framework incorporates each of the Countdown-specific domains that will contribute to or detract from progress in RMNCAH: health systems and policy, health financing, coverage, equity and mortality (including the Lives Saved Tool). While we have previously examined coverage, equity and mortality, the current paper will address the remaining domains of health systems, policies and financing over the MDG period. The conceptual framework reflects our broader objectives of understanding RMNCAH change in Kenya; thus, some aspects of maternal and newborn health are reported within these domains, despite the paper’s focus on child health.

### Mortality trends and coverage estimates

Raw data from nationally representative Kenya Demographic and Health Surveys (K-DHS) from 1989 to 2014^2 5–9^ were used to generate under-five mortality estimates over the MDG period (1990–2015). For simplicity of interpretation, we refer to the year of the survey (1993, 2003 and 2014) in our results, though U5MR estimates actually reflect child mortality for the survey’s preceding 5-year period. Coverage indicators were defined as per the Countdown to 2015 guidelines.^10^ A Composite Coverage Index (CCI) was calculated to present an overall picture of intervention coverage in Kenya^11^ (online supplementary etable 1). CCI is comprised of the following set of eight preventive and curative interventions: (1) demand for family planning satisfied, (2) skilled birth attendance, (3) antenatal care with a skilled provider (ANC), (4) DPT3 vaccination, (5) measles vaccination, (6) BCG vaccination, (7) oral rehydration therapy and continued feeding for children with diarrhoea and (8) care seeking for children with suspected pneumonia. Use of CCI as a summary statistic is a novel feature among standardised Countdown country case studies. K-DHS 2014 was powered at the county level, while all previous DHS surveys are powered for provincial estimates. Statistical packages R and Win bug 14 were used to estimate prevalence, and ArcGIS10 was used to create high-resolution maps for visualisation of CCI across counties.

### Health systems and policies

To assess policy and systems changes relevant to RMNCAH in Kenya from 1990 to 2014, we used the following three standardised tools developed by the Countdown Health Systems and Policies Technical Working Group and applied in previous Countdown case studies^12–14^: (1) Policy and Program Timeline Tool; (2) Health Policy Tracer Indicators Dashboard; and (3) Health Systems Tracer Indicator Dashboard.^4^ Data for these tools were primarily populated through the review of existing published and grey literature, including peer-reviewed publications, Ministry of Health (MoH) policy and strategy documents, MoH reports, WHO and United Nations agencies reports and databases, for example, data from the WHO Global Maternal, Newborn, Child and Adolescent Health policy indicators database.^15^ Health workforce and facility data were obtained from the 2004 Service Availability Mapping survey^16^ and the most recent WHO Service Availability and Readiness Assessment Mapping for Kenya.^17^ Spider plots were used to visually depict status of policy implementation, whereby each major policy was dissociated into related subpolicies, and implementation level (%) was mapped within the plot. Spider plots were based on up-to-date data from the WHO database.^15^ To examine the relationship between health systems inputs and service accessibility and delivery, we overlaid health systems data (health workforce, health facility density, health services budget and lifesaving commodities) on a map of CCI. We also conducted key informant interviews with stakeholders from the MoH, Division of Family Health, WHO and the World Bank as part of the methodology for populating, verifying and analysing data in the Policy and Program Timeline Tool.^4^

### Health financing

For the health financing analysis, we undertook a review of the existing literature to describe the evolution of health financing policy in Kenya. Information from National Health Accounts (NHA) for fiscal years 2005/2006, 2009/2010 and 2012/2013,^18^ *Health Facts and Figures 2014* 19 compiled by the Ministry of Health, the Countdown to 2015 database on official development assistance (ODA) for RMNCAH^20^ and the 2003 and 2013 Kenya Household Health Expenditure and Utilisation Survey^21 22^ were used in order to determine trends in health spending.

### Multivariable analysis

Multivariable analyses were performed to determine factors associated with improvements in child survival across the 1993–2014 MDG period. We evaluated data from three K-DHS surveys (1993, 2003 and 2014) to reflect the early (1990–1999), mid (2000–2009) and late (2010–2015) MDG periods. Robust and representative U5MR estimates were available for these survey years. Maternal mortality was variable and available for only two time points (2003 and 2014), and neonatal mortality did not sufficiently decline to perform a robust analysis of change. Due to the nested nature of the K-DHS data, mixed-effects, multilevel logistic regression models were used. Hierarchical model building strategies were used to determine multivariable predictors of U5MR.^23^ Using evidence-based child survival frameworks,^24 25^ variables were mapped into two levels that corresponded to intermediate (level 2) and proximal (level 1) determinants of child mortality. Intermediary factors included geographical region, residence, access to improved water and sanitation facilities, maternal and paternal education and household wealth index, a composite measure based on household assets. At the proximal level, we examined individual child and parent characteristics including gender, birth order, birth interval, size at birth, timing of breastfeeding initiation, age of mother at birth, contraceptive use and parity. For each survey (1993, 2003 and 2014), the dependent variable was U5MR, while the independent variables encompassed all intermediate and proximal determinants listed above. Bivariate associations between the determinant and U5MR were assessed using ORs and corresponding p values/95% CIs. Factors statistically significant at p<0.20 were entered into multivariable modelling within the respective level and retained if p<0.15. Multicollinearity was assessed using variance inflation factors (VIF), where variables with VIF >3 were considered collinear.^26^ Additionally, a correlation matrix for all predictive variables is appended (online supplementary eTable 2). A secondary analysis was conducted to determine the effect of change in the determinants on change in U5MR between surveys. These methods are based on similar analyses by Hong *et al* 27 conducted using DHS data from three time points in Rwanda. Changes in the distributions of indicators between surveys (2014 compared with 2003 and 2014 compared with 1993) were multiplied by the coefficients of variables obtained from the final 2014 multivariable model of mortality, as described above. The products were then summed, and the results were exponentiated to obtain the change in mortality that was due to changes in the values of the indicators between surveys.^27^ In other words, this procedure allows you to determine how much of the change in child mortality from 1993 to 2014 is due to shifting proportions of the explanatory variables between surveys.

To account for the impact of the HIV/AIDS epidemic on child mortality in Kenya, we have included a dedicated section on HIV/AIDS trends. The first Kenya Aids Indicator Survey (KAIS) took place in 2007, after the peak of the HIV epidemic in Kenya. Before KAIS, the 2003 K-DHS was the first national, household-based survey to include HIV testing.^8^ As such, we used both K-DHS data and modelled data from the Joint United Nations Programme on HIV/AIDS (UNAIDS)^28^ to report HIV epidemic trends throughout the MDG period in Kenya (1990–2015). Coverage of antiretroviral treatment (ART) and indicators relating to prevention of mother-to-child transmission were not available from UNAIDS until 2010.

### Role of the funding source

The funders of the study had no role in study design, data collection, data analysis, data interpretation or writing of the report. The corresponding author had full access to all the data in the study and had final responsibility for the decision to submit for publication.

## Results

### Health systems and policy

**Figure 1:**
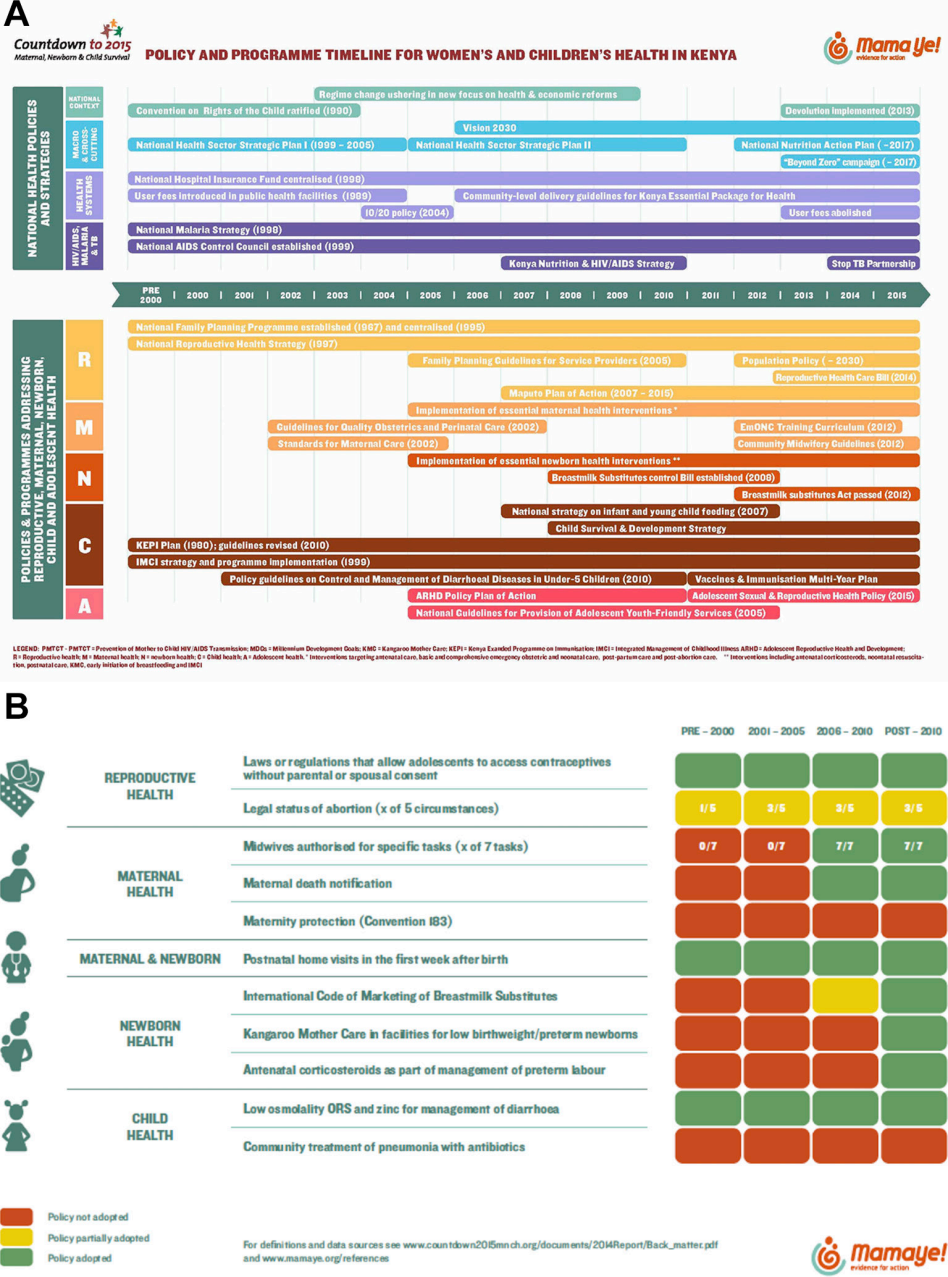
(A) Health systems and policy timeline for national-level and RMNCAH-specific policies and programmes. (B) Policy tracer indicators for women’s and children’s health in Kenya.

Results from the Policy and Program Timeline Tool (figure 1A) show a complex policy environment since 1990. Following a crisis of health and social development in the 1980s, an intensive restructuring of the Kenyan healthcare system in the early 1990s led to the publication of the Kenya Health Policy Framework Paper in 1994, which outlined reform in key areas that focused on sustainable, accessible and affordable quality healthcare. With decentralisation as the guiding strategy, the policy framework was implemented through two 5-year plans: the National Health Sector Strategic Plan (NHSSP) I (1999–2004) and II (2005–2010). However, an evaluation of NHSSP I revealed that it did not meet significant targets and indicators of health and socioeconomic development based on mortality rates, health service utilisation, health workforce density, out-of-pocket health spending and poverty levels^29^; findings that are consistent with evidence from this time period reporting increased U5MR in Kenya.^3^ Vision 2030, a long-term national development plan based on economic, social and political pillars, was launched in 2006. The health sector defined a central theme of the social pillar; related priorities centre on restructuring of leadership and governance, improving procurement and availability of essential medicines, modernising information systems, accelerating infrastructure and ensuring equitable access. However, recurring challenges to the implementation of each of these plans include disparities in access between the urban, rural and hard-to-reach areas, inadequacy of health infrastructure across the country and shortages of human resources for health. Financial bottlenecks include limitations in the availability of fiscal resources alongside the high cost of accessing healthcare for much of Kenya’s population. With these barriers in mind, NHSSP II proposed to accelerate decentralisation of quality health services to rural areas, a factor also reinforced through the New Constitution and devolution from 2013.^30^ It was envisaged that decentralisation of health services to the county would enhance the quality of care, equity and efficiency in service delivery, helping Kenya to achieve its health targets. So far, this transition has been beset with several technical, logistical and sociopolitical challenges, particularly surrounding capacity gaps in management, tracking of health spending by programme and equitable distributions of human resources for health.^31–33^

Despite consistent commitment to health in national plans and strategies since 1990, results show that RMNCAH-specific interventions and targets multiplied in 2005, aligning with the period where maternal and child health gained significant global attention. However, important policy and programmatic differences were noted across the RMNCAH continuum (figure 1A). Compared with reproductive, maternal and newborn health programmes, child health programmes have been implemented consistently since the 1990s, in particular those delivered at the primary care level, such as the Kenya Expanded Programme on Immunization. Reproductive and maternal health visibility began in the 1980s, with variable and inconsistent programme implementation. In contrast, newborn health is absent from the policy timeline before 2005 (figure 1A), with full adoption of most related policies not occurring until the post-2010 era. The policy indicator tracer dashboard (figure 1B), a tool that is used to monitor standardised, Countdown-specific policy indicators, shows similar progress to the timeline, with adoption of most policies occurring after 2005. Figure 1B highlights the considerable steps that Kenya has taken to implement critical RMNCAH policies but also underscores the gaps that remain in the adoption of some, including the circumstances for legal abortion, maternity protection of employed women (Convention 183) and community treatment of child pneumonia with antibiotics. In fact, when looking further at policies relevant to Integrated Community Case Management of child illness (online supplementary eFigure 2), several have been only partially adopted (management of pneumonia in community/home and community based providers trained to manage pneumonia, diarrhoea and malaria in an integrated manner) or not adopted at all (paid community-based providers for pneumonia, diarrhoea and malaria care). Considering that pneumonia, diarrhoea and malaria are the leading causes of death for children under 5 years of age in Kenya,^3^ appropriate and integrated treatment of these conditions that goes beyond health facility-based services will be imperative to reduce child mortality.

The current administration has undertaken several initiatives to address the gaps in healthcare that remain, including implementation of the Free Maternity Services policy in all public health facilities in 2013.^34 35^ Preliminary results in 2014 revealed improvements in several important indicators, such as greater attendance of at least four antenatal care visits and increased HIV testing and counselling during pregnancy.^35^

### Health workforce

**Figure 2:**
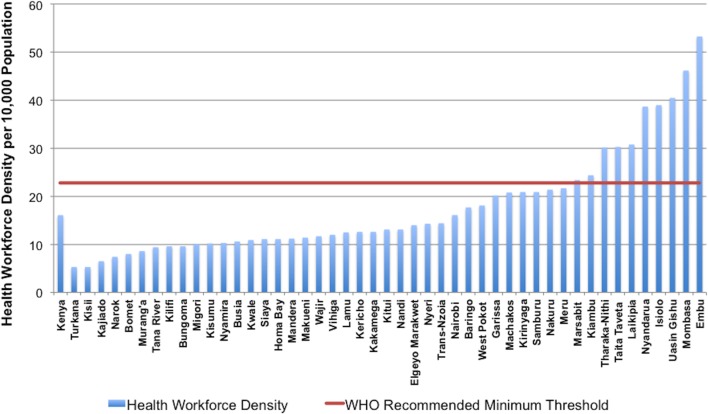
Health workforce density by county (2013).

Overall, there is a high correlation between health workforce density, service delivery and health outcomes.^36^ Since 1990, Kenya’s health workforce has been insufficient to meet population needs. Results from Kenya’s first assessment in 2004 indicated a health workforce density (including all doctors, clinical officers, nurses, laboratory technicians, pharmacists, health records personnel and information officers) of 16 per 10 000 in Nairobi and 6.9 per 10 000 for the rest of the country.^16^ Recent estimates for Kenya reveal an average of 17.7 healthcare workers per 10 000 people.^17^ However, when disaggregating these data by county (figure 2), significant regional disparities are noted, with only 10 of 47 counties meeting the WHO minimum density threshold of 22.8 workers per 10 000 population.^36^

**Figure 3:**
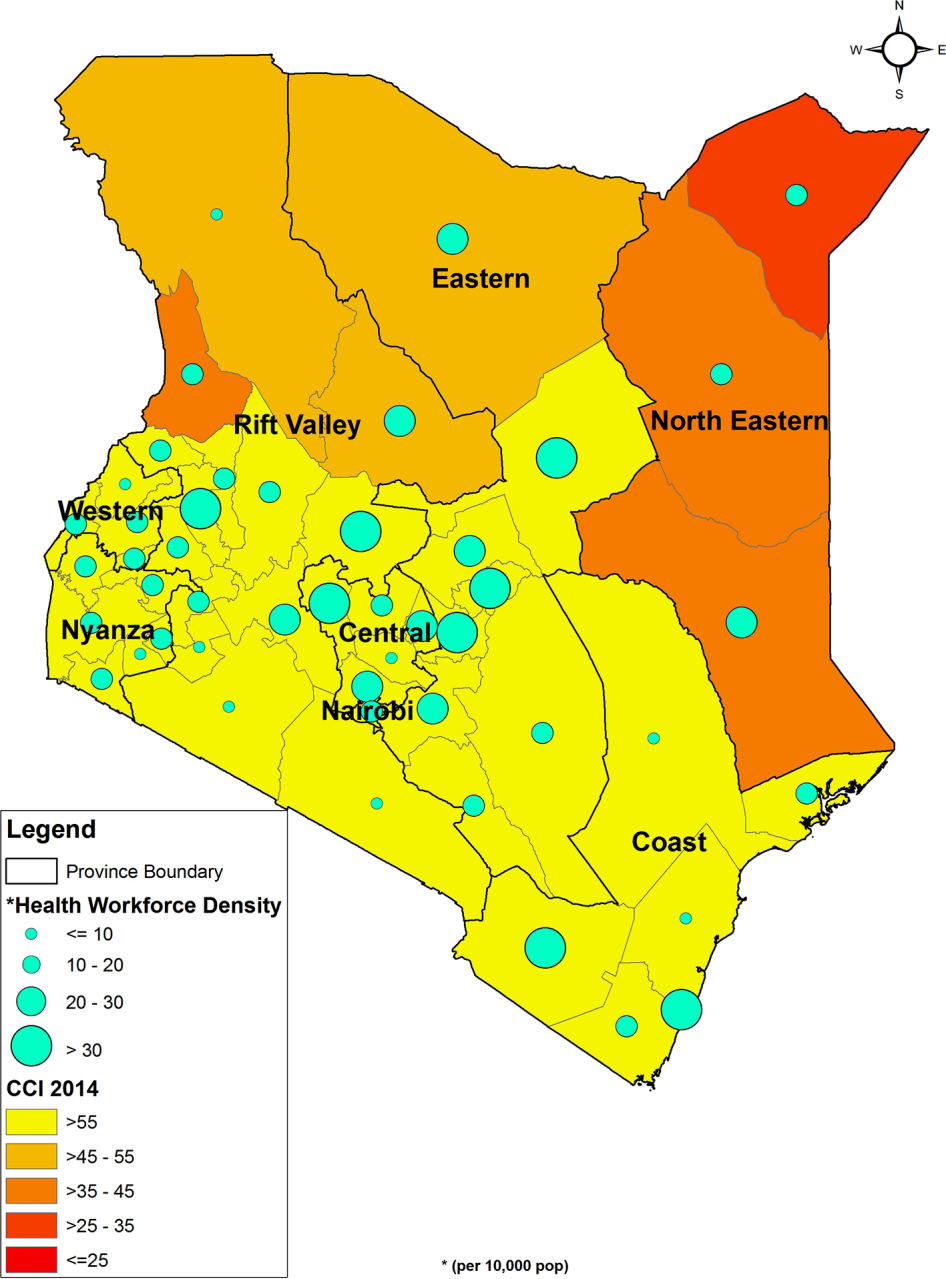
Health workforce density and CCI (2014). CCI, Composite Coverage Index.

Figure 3 indicates that in regions where health workforce density is below the recommended WHO minimum threshold, there is also low intervention coverage. Within this map, county-level CCI ranges from less than 25% to greater than 55%, with Mandera, Wajir, Garissa and West Pokot counties faring the worst. Though workforce density is variable throughout the country, counties comprising Nairobi, Central and part of the Eastern regions have the highest health workforce density. Additional mapping of CCI and health facility availability of life-saving commodities (online supplementary eFigure 3) reveals a similar pattern, whereby regions with low CCI coverage also demonstrate a lack in supply of essential medicines (availability ranging from <25% to 35%).

### Health financing

Results from the financing analysis demonstrated an inconsistent approach to health financing in Kenya, including financing for critical RMNCAH services. Following independence in 1963, the Government of Kenya (GoK) formalised the concept of free healthcare for all. However, with the growing population and worsening socioeconomic and political factors, a severe crisis of health and social development unravelled in the late 1980s, and GoK was forced to implement a cost-sharing scheme in 1989^37^. This approach levied user fees for all public health facilities and at all levels of care to offset operating costs.^38 39^ Recognising that this would depress utilisation, GoK initiated waivers and exemptions for priority interventions and populations, including children under 5 years of age. Nonetheless, several studies have since shown that the introduction of user fees was detrimental to health service accessibility, with the poorest experiencing the greatest financial barrier to access.^40 41^ Adhering to their political pledge, the National Rainbow Coalition government promulgated several high profile policies to address financial barriers associated with accessing healthcare services following the change in regime in 2003. These included the 10/20 policy of 2004, where a flat registration fee of 10 and 20 KES replaced user fees at dispensaries and health centres, respectively.^39^ However, the Public Expenditure Tracking Survey of 2012 found that only 45% of facilities complied with the 10/20 policy, despite implementation being reported by 86% of facilities, indicating that user fees continued to account for the majority of facilities’ operating budgets.^42^ In 2013, the current administration announced a policy for the removal of user fees at all government-owned primary care facilities and free maternity services at all public facilities.^37^ However, this policy was introduced as Kenya commenced the process of devolution, whereby the responsibility for a range of health functions, including the delivery of primary and secondary care, was shifted from the national government to newly formed county governments. This has given rise to host of operational challenges for the new user fee removal policies, such as delays in intergovernmental financial flows, non-uniform implementation of the policy across different countries and weak county structures facing difficulties in meeting increased demand for maternity services at public facilities.^42^

Between the years 2001 and 2013, total health spending in Kenya doubled from KES 109 billion (US$1.4 billion) to 234 billion (US$2.7 billion) (online supplementary eFigure 4a) after adjusting for inflation.^18^ While the share of total health spending that is financed from public resources grew modestly from 29.6% to 33.5%,^18^ the government’s allocation to health as a share of total government expenditure has decreased (online supplementary eFigure 4b).^19^

Insurance coverage rose from 10% of the population in 2003 to a mere 17% by 2013.^21 22^ Of those insured, 88% were members of the National Hospital Insurance Fund (NHIF),^21^ a government run insurance scheme that historically focused on hospitalisation costs for employees of the formal sector. In recent years, NHIF has made a push to grow voluntary membership from the informal sector and expand its benefit package to include outpatient services.^43^ A much smaller fraction of typically wealthier Kenyans have private insurance. In contrast, out-of-pocket expenditure (OOPE) accounted for one quarter of total health expenditure in 2012/2013.^18^ The OOPE per visit for an outpatient visit was 383 KES (US$4.5).^21^ However, in a country where nearly 40% of the population spends less than US$2 a day, even this modest amount can pose a significant barrier to healthcare utilisation. Indeed, the high cost of care was among the top three reasons cited by survey respondents for foregoing care when they were sick.

**Figure 4:**
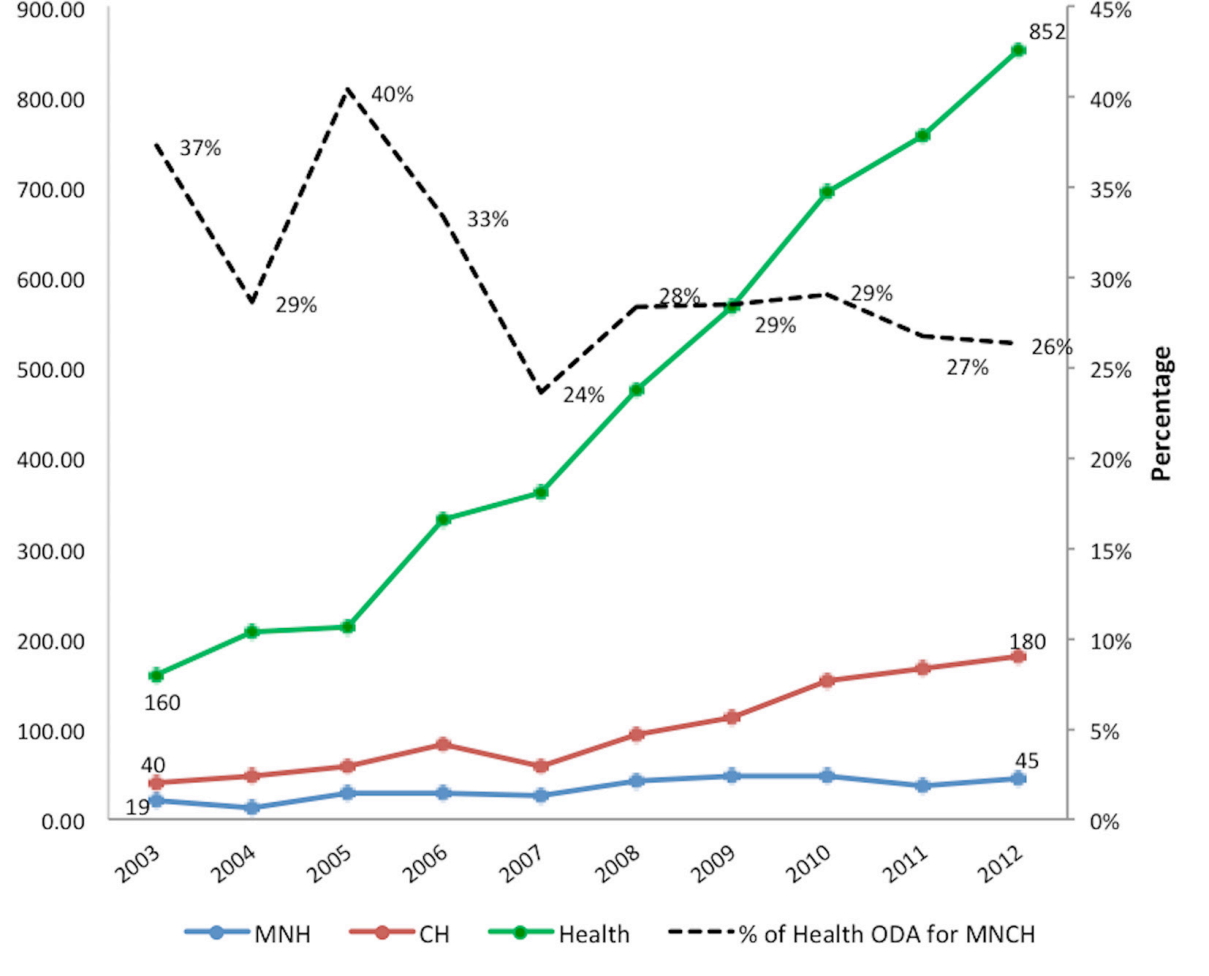
Trends in health spending and ODA for health from 2003 to 2012. ODA, official development assistance. CH, child health; MNH, maternal and newborn health.

While donor financing for health increased dramatically between 2003 and 2012, only about 25% was allocated for RMNCAH in 2012, down from a period high of 40% in 2005 (figure 4). Aid for child health programmes quadrupled from US$40 million in 2002 to US$180 million in 2012 after adjusting for inflation, but external investments for maternal and neonatal health remained low, increasing from US$19 million to US$45 million over the same period^20^ (figure 4).

### Factors associated with decline in child mortality

Beyond macro-level systems and finance transitions in Kenya, survival of women and children may have improved due to changes in underlying proximal and intermediary determinants of mortality. Using U5MR as the outcome, we explored these factors further. In absolute terms, U5MR declined by 50%, from 93.2/1000 in 1993 to 51.3/1000 in 2014^3^. However, the greatest decline was observed between 2003 and 2014, after a peak in U5MR of 115.0/1000 in 2003.

As such, we explored correlates of U5MR for three time points (1993, 2003 and 2014) to further understand changes in the proximal and intermediate determinants of child survival in Kenya across early stage, midstage and late stages of the MDG commitment. The trends in child mortality, rising throughout the 1990s and falling thereafter, lend themselves to the design of these analyses. Full crude and adjusted models for 1993 and 2003 are detailed in the online supplementary material. In adjusted analyses, several intermediate-level contextual determinants were associated with child survival in 1993 and 2003, including the geographical area of residence, maternal and paternal education and household income (online supplementary eTable 3). More proximally, shorter birth intervals, small size at birth, lack of early breast feeding within 1 hour, higher parity and maternal obesity were significantly associated with higher U5MR in 1993. By 2003, additional factors had gained prominence in their relation to poor child survival in adjusted analyses, including the child being a male, higher birth order and lack of contraceptive use among mothers (online supplementary eTable 4).

Despite several potential determinants in crude analyses, only maternal education, household wealth index and geographical region of residence were significantly related to U5MR in 2014, in a model that also adjusted for rural residence (online supplementary eTable 5). As expected, mortality was higher among children born to less educated mothers and those from poorer households. Interestingly, relative to families living in Nairobi, those in any other region in Kenya, had lower odds of child mortality. Children in the North East (OR=0.41, 95% CI 0.25 to 0.69), Rift Valley (OR=0.44, 95% CI 0.29 to 0.67) and Eastern regions (OR=0.47, 95% CI 0.30 to 0.73) had the lowest odds of death relative to those living in Nairobi. Small size at birth, lack of early breast feeding, low contraceptive use, higher parity, poor access to improved sanitation facilities and failure to attend ANC during pregnancy were associated with higher U5MR in adjusted analyses (p<0.15) (eTable 5).

We analysed factors that were associated with the decline in U5MR from 1993 to 2014 (table 1). Between 1993 and 2014, U5MR in Kenya decreased from 96.1 to 52.0 per 1000 live births—a 46% reduction. This was significantly positively related to a higher maternal literacy, increases in household income, population shifts (out of Nairobi and into Rift Valley and Coast regions), decreases in fertility rates, decreased prevalence of small-sized babies, higher prevalence of early breast feeding, higher contraceptive prevalence and lower parity. Collectively, these factors accounted for almost one-third of the decline in U5MR from 1993 to 2014 [(13/46)×100=28%]. The decline over this period was offset by an increase in the proportion of families without access to improved sanitation facilities and a slight increase in mothers not seeking ANC. Transitions in prevalence of key contextual determinants, health conditions and interventions were accelerated in the 2003–2014 time period; interpretations can be made analogously as above. Between 2003 and 2014, the U5MR declined by 55% ((115–52)/115), and the mentioned determinants are responsible for 44% ((24/55)×100)) of this decline (table 1).

**Table 1:** Factors associated with decline of U5MR in Kenya, K-DHS 1993, 2003 and 2014

Indicators	Proportions			Change in proportions		Model (2014)		U5MR by change in proportions	
	1993	2003	2014	2014–2003	2014–1993	Coef.	P values	2014–2003	2014–1993
Intermediate level factors									
Maternal education (ref: secondary and above)									
No formal education	0.1917	0.1537	0.1179	−0.0358	−0.0738	0.339	0.028	−0.0121	−0.0250
Incomplete primary	0.3948	0.3642	0.2853	−0.0789	−0.1095	0.344	0.006	−0.0271	−0.0376
Primary	0.3904	0.3605	0.3796	0.0191	−0.0108	0.124	0.291		
Wealth index (ref: richest)									
Poorest	0.2289	0.2474	0.238	−0.0094	0.0091	0.208	0.191		
Poorer	0.2134	0.2082	0.2038	−0.0044	−0.0096	0.277	0.073	−0.0012	−0.0027
Middle	0.1962	0.1899	0.1802	−0.0097	−0.0160	0.358	0.018	−0.0035	−0.0057
Richer	0.1939	0.1692	0.1765	0.0073	−0.0174	0.225	0.121	0.0016	−0.0039
Type of place of residence (ref: urban)									
Rural	0.8728	0.8127	0.641	−0.1717	−0.2318	−0.143	0.113	0.0246	0.0332
Region (ref: Nairobi)									
Central	0.1147	0.1069	0.0918	−0.0151	−0.0229	−0.427	0.07	0.0065	0.0098
Coast	0.0893	0.0836	0.1034	0.0198	0.0141	−0.545	0.014	−0.0108	−0.0077
Eastern	0.2017	0.1551	0.1187	−0.0364	−0.0830	−0.759	0.001	0.0276	0.0630
Nyanza	0.1669	0.1639	0.1426	−0.0213	−0.0243	−0.274	0.209	0.0059	0.0067
Rift Valley	0.2167	0.2685	0.2902	0.0217	0.0735	−0.823	0	−0.0179	−0.0605
Western	0.1651	0.1271	0.1153	−0.0118	−0.0498	−0.561	0.016	0.0066	0.0280
North Eastern	–	0.0296	0.0332	0.0036		−0.881	0.001	−0.0032	0.0000
Sum								−0.0029	−0.0024
exp(sum)=rel. risk								0.9971	0.9976
Proximal level factors									
Improved sanitation facility (ref: yes)									
No	0.1219	0.1397	0.2092	0.0695	0.0873	0.423	0.109	0.0294	0.0369
Fertility (ref: 1–2 children)									
3–4	0.281	0.3259	0.3156	−0.0103	0.0346	0.842	0	−0.0087	0.0291
≥5	0.4196	0.3279	0.2638	−0.0641	−0.1558	0.398	0.092	−0.0255	−0.0620
Contraceptive use (ref: yes)									
No	0.7092	0.6775	0.7397	0.0622	0.0305	−0.621	0.001	−0.0386	−0.0189
ANC visits (ref: 4+ visits)									
No ANC	0.0514	0.122	0.0529	−0.0691	0.0015	0.963	0.002	−0.0666	0.0014
<4 visits	0.3972	0.4532	0.5088	0.0556	0.1116	0.0410	0.838		
Early initiation of breast feeding (ref: <1 hour)									
>1 hour	0.384	0.417	0.273	−0.1440	−0.1110	1.094	0	−0.1576	−0.1215
Size at birth (ref: average)									
Small/very small	0.1576	0.1637	0.1516	−0.0121	−0.0060	0.410	0.069	−0.0050	−0.0025
Large/very large	0.3195	0.2542	0.2593	0.0051	−0.0602	0.146	0.498		
Sum								−0.2725	−0.1374
exp(sum)=rel. risk								0.7614	0.8716
ALL (level 2+level 1)									
Overall									
Sum								−0.2755	−0.1398
exp(sum)=rel. risk								0.7592	0.8695

ANC, antenatal care; coef., beta coefficient; exp, exponentiated; K-DHS, Kenya Demographic and Health Surveys; rel. risk, relative risk; U5MR, under five mortality rate.

This model explains 28% and 44% of the decline in U5MR noted for the complete and the latter half of the MDG period. While our modelling strategy was robust and complete in terms of available household and individual level variables, it indicates that other factors may have contributed to U5MR decline.

### HIV/AIDS in Kenya

One factor in particular that was not accounted for when interpreting trends in child mortality was the HIV/AIDS epidemic that coincided with the Countdown study period. National HIV prevalence among adults aged 15–49 years was 5.9% (4.8–7.4) in 1990, equating to 690 000 (560 000–870 000) people living with HIV (online supplementary eFigure 5a). This peaked at 11.1% (9.6–12.8) in 1997, at which point the prevalence reduced to 7.4% (6.6–8.2) in 2005 and further to 5.6% (4.9–6.3) in 2015. In 2015, an estimated 1 600 000 (1 400 000–1 700 000) adults in Kenya were living with HIV. Among children aged 0–14 years, an estimated 40 000 (30 000–52 000) had contracted HIV by 1990, mostly through mother-to-child transmission. This figure rose to 220 000 (180 000–260 000) by 2003 and has since reduced to 130 000 (110 000–160 000) children who were estimated to be living with the disease in 2015. The number of AIDS-related deaths in children reached a peak of 24 000 (19 000–28 000) deaths per year by 2002. AIDS-related child mortality was mainly due to direct effects of the disease, as a consequence of mother-to-child transmission, but also encompassed indirect effects, including illness and death of caregivers (leading to an explosion of AIDS orphans), increased opportunistic infections in areas of high HIV prevalence and poor economic and household conditions as a consequence of the epidemic. By 2015, the number of AIDS-related deaths per year in children had declined to 5700 (3600–7900).

Several regions were hit harder than others, in particular Nyanza and Nairobi. Modelled subnational estimates for the year 2000 found a prevalence of 19% in Nyanza province, 11% in Nairobi province and 10% or under for the remaining six provinces (online supplementary eFigure 5a). The 2003 K-DHS corroborated these findings, reporting a prevalence of 15% for Nyanza province followed by 10% for Nairobi and 4%–6% for all other provinces. The 2003 K-DHS data also revealed that urban residents had a higher risk of HIV infection compared with rural residents (10% vs 6%).

Although we were not able to include HIV-related variables within our multivariable modelling, the trends that we have outlined here underscore the substantial contribution of the HIV/AIDS epidemic to under-five mortality in Kenya. In fact, overlaying U5MR data with the number of AIDS-related deaths in children from 1990 to 2015 demonstrates an analogous pattern (online supplementary eFigure 5b). According to the Child Health Epidemiology Reference Group, which provides estimates specifically for children under 5 years of age, AIDS-related child deaths declined by 59% between 2000 and 2010.^44^ Presumably, some of the decline in U5MR was due to the substantial response with ART, though good quality data to monitor ART coverage was not available until the 2007 KAIS. However, estimates from annual HIV/AIDS progress reports found that trends in the percentage of HIV-positive pregnant women and exposed infants receiving ART for prevention of mother-to-child transmission improved from 24% and 20%, respectively, in 2004 to 73% and 49% in 2009.^45^

## Discussion

Our review of Kenya’s rapidly changing contextual landscape suggests that improvements in maternal and child health and survival are linked to multifaceted and multisectoral national and subnational efforts. Though child mortality declined by about half from 1993 to 2014, the insufficient health workforce and lack of focus on RMNCAH pre-2000 contributed to a detrimental rise in preventable child deaths from 1993 to 2003 (wherein U5MR increased by 23%). Strong national commitment driven by global MDG priorities for improving RMNCAH post-2000 accelerated the 55% decline in U5MR from 2003 to 2014. Major determinants of child survival were the decrease in fertility/greater birth spacing, improvement in pregnant mothers’ and newborn nutrition, household socioeconomic factors and geography. Additionally, health spending in Kenya doubled between 2001 and 2013, with donor financing for child health interventions quadrupling over the same period. Aligning with the increased state funding for child health, policies and programmes aimed at reducing child deaths through the prevention and control of major childhood illnesses expanded; both contributed to the decline in mortality noted for the latter half of the MDG period.

We performed a robust multivariable analysis that used individual-level data to examine the determinants of child survival across the MDG period. Our model was able to account for approximately 30% of decline in U5MR from 1990 to 2014 and 45% of mortality decline for the period 2003–2014, though it had several limitations to note. We did not examine mortality trends at the level of the county because of scarcity of data and survey power at that level of specificity. Because the data are both cross-sectional and retrospective, covariates may pertain to the time of birth or time of survey, as opposed to the time of the child’s death. As such, we caution the reader of this time lag when interpreting the multivariable results (ie, deaths are representative of the five preceding survey years, while certain explanatory variables have been measured at the time of the survey). However, to overcome this limitation, we have used the change, or difference, in covariates between two surveys (eg, between 2014 and 1993 and between 2014 and 2003) as the predictors. Several key indicators of child health were restricted to a 2-week recall of living children (eg, presence of diarrhoea or pneumonia and care seeking for these illnesses) and could not be included in this analysis of mortality. Many of the variables used in our analysis were limited to those found within the K-DHS datasets and, as such, did not include important distal determinants such as governance (eg, political stability and state autocracy/democracy), conflict (eg, terrorism incidents and refugee populations), environment (eg, drought and natural disasters) and infrastructure (eg, urbanisation). Recent efforts by Burke *et al* 46 used a powerful geospatial modelling approach to compare sources of variation in U5MR between and within 28 countries in sub-Saharan Africa using contextual and relevant data from DHS surveys. These authors found that mortality was driven by subnational factors including temperature, malaria burden and conflict.^46^ Another multicountry study across 146 low-income and middle-income countries found that improvements within the health sector accounted for only 50% of maternal and child mortality reductions from 1990 to 2010, while the other half were due to gains outside the health sector, including those related to literacy, income, gender equality and the environment.^47^ These important findings resemble ours and highlight the wisdom that informed the structuring of the SDGs as a multisectoral effort with health at the centre.

Importantly, we were unable to examine impact of the HIV/AIDS epidemic on U5MR throughout the MDG period (due to lack of related K-DHS data), though it is known to be an important determinant in Kenya. In an effort to highlight this, we have provided an overview of HIV epidemic trends over the period, including regional and socioeconomic disparities in HIV infection risk, many of which paralleled our findings on the determinants of U5MR. For example, adjusted analysis revealed a 2.0 and 2.3 times higher risk of child mortality in Nyanza when compared with Nairobi in 1993 and 2003, respectively. Additionally, we found that the decline in child mortality over the period from 1993 to 2014 was impacted by region, with risk of mortality being higher in Nairobi, a finding that may be reflective of urban/rural differentials in HIV prevalence. Other studies in Kenya have demonstrated a strong association between HIV/AIDS and the increase in U5MR noted during the early half of the MDGs,^48 49^ though few have investigated the HIV/AIDS response as it relates to child mortality decline. Taken together, our analysis does suffer from omitted variable bias. This type of bias can occur when a variable that interacts with both the explanatory and outcome variables in a model is missing, such that the estimated effects of other variables on the outcome is biased upwards or downwards. When interpreting the results of our multivariable analysis, it is important to keep in mind the potential over-representation of impact from other factors due to the missing HIV variable.

There are additional challenges that accompany performing a rigorous health systems and policy (HSP) analysis. Often, there is poor tracking of the policy formulation to implementation pathway in LMIC settings because of unstandardised HSP definitions and tools,^4^ and currently, there is no consensus on how to appropriately measure implementation intensity relating to RMNCAH interventions.^50^ Within our assessment, we were unable to accurately capture implementation strength, or whether a policy was fully implemented, partially implemented or not implemented at all. Our analyses would be further strengthened if complemented with analyses of governance, power and partnerships to fully understand how and why policy change took place. Although we used standardised tools to assess policy change over time and by programme at the national level, the HSP assessment needs further development at subnational level, particularly to better understand variation in RMNCAH outcomes. As HSP measurement methodologies are improved and standardised, we will be able to better understand which programmes, policies and systems changes will achieve the most future health gains. Like the HSP component, the health financing analysis adopted a mixed-methods approach based on data from surveys, government documents and stakeholder interviews. As such, we were able to glean a comprehensive understanding of the health financing landscape in Kenya, including some of the regulatory tools used to increase uptake of priority interventions. However, the data are slightly outdated, with the most recent NHA being reported for fiscal year 2012/2013 and ODA reflecting aid in 2013. Additionally, we do not have detailed estimates of health spending by county governments on specific programme areas because the counties are currently not using program-based budgeting. This makes tracking of postdevolution RMNCAH-specific spending extremely challenging.

A major finding of this study was the geographic differentials in mortality, with Nairobi being a significant determinant of U5MR when compared with all other regions in Kenya. Other recent studies have also found lower declines in childhood mortality in urban areas when compared with rural areas,^51 52^ indicating that the once acclaimed ‘urban advantage’ has since narrowed or reversed. This aligns with K-DHS data that place Nairobi as having the second highest U5MR in 2014 (72 per 1000 live births).^2^ Though not conclusive, one such explanation for this finding could be related to urban growth, resulting in large sections of the population living in informal settlements or urban slums. Slums are characterised by overcrowding, insecure tenure, poor water and sanitation, high rates of HIV and other infections and inadequate health services.^53 54^ In these densely packed neighbourhoods, children are especially vulnerable. In 2007, 72% of the urban population in sub-Saharan Africa resided in slums or slum-like conditions,^55^ a number that has likely worsened given the current refugee crisis. In Nairobi alone, there are over 100 slums that occupy between 60% and 70% of its population of 3 million.^55^ Consistent with trends that accompany rapid urbanisation, we found that the proportion of families without access to improved sanitation increased over the MDG period, and this offset the decline in U5MR. Others have demonstrated that household characteristics, including access to clean drinking water, sanitation facilities and low polluting fuels for cooking, are significantly associated with lower U5MR.^56 57^ Taken together, more local research is required for planning of low-income urban settlements, including how to improve the built environment and how to appropriately target healthcare services to better reach these populations.

Another unique finding of our multivariable analysis was the clear link between family planning and child mortality. Higher contraceptive use, lower parity and decreased fertility were each positively associated with the decline in U5MR between 1993 and 2014. The reasons behind this are likely to be multifaceted. First, family planning promotes gender equity through choice, providing women the ability to control their fertility and time their pregnancies. It helps girls remain in school, reduces poverty and unequivocally saves lives.^58 59^ Use of contraception leads to reductions in pregnancies for at-risk groups, such as adolescents, improving maternal, perinatal and child survival and reducing unsafe abortions.^60^ Projections from 2015 estimate that if Kenya were to achieve its family planning goals by 2020, more than 2000 maternal deaths could be avoided and 850 000 unintended pregnancies could be prevented over a 5-year period.^61^ Its impact on under-five mortality has both direct and indirect associations. For example, spacing of pregnancies has been shown to improve birth and nutritional outcomes, including reducing the risk of prematurity, low birth weight, small for gestational age and stunting.^62 63^ Birth spacing also directly reduces infant and child deaths. Analyses have suggested that an interval of less than 2 years can raise the risk of child mortality by 40%.^60 64^ Family planning is a simple and cost-effective intervention that has major transformative potential for women and families in low-income and middle-income settings. Following the Family Planning Summit in 2017, GoK has committed to improving family planning services in Kenya through several means. The portion of national budget allocated directly to family planning has increased, efforts have been made to improve the availability of long-acting and permanent methods of family planning in both the public and private sector and service provision for adolescents and youth has become a major focus.^65^

Along with enabling the environment for improved family planning, GoK has renewed its commitment to advancing RMNCAH in Kenya through several other bold initiatives, including partnering with the Beyond Zero Foundation. Spearheaded by the first lady of Kenya, the objective of the Beyond Zero campaign is to reduce mortality through improved access to quality RMNCAH services.^66^ To date, 47 fully equipped mobile clinics have been distributed to each devolved county unit to provide integrated HIV, and maternal and child health outreach services to those in need. Additionally, the Transforming Health Systems for Universal Care Project aims to improve primary care for women of reproductive age, including adolescents, and children under 5 years of age by expanding delivery and access to health services within the county, along with institutional capabilities.^67^ To do this, county governments will focus on the scale-up of evidence-based interventions along the continuum of care, improving quality of services, strengthening monitoring and evaluation and working towards health financing reform to achieve universal health care.^67^ These initiatives will help to build the capacity of health systems in the devolved counties and will support Kenya’s pledge to achieve SDG target 3 (to ensure healthy lives and promote well-being for all at all ages) through attaining universal health coverage. Despite improved outreach services, Kenya’s public healthcare system has suffered over the 2017 year, plagued by repeated doctors’ and nurses’ strikes that have left untreated patients vulnerable. Along with better wages and working conditions, a major demand by healthcare professionals was the need to address the severe shortage of doctors and nurses across the country. There is hope that the government can work to better address these critical health systems issues following the recent presidential election. In fact, all Kenyans, including those in government, the health sector, civil society and in communities, need to ensure that the health agenda is pushed forward in order to better protect the country’s growing population.

The results of this analysis have demonstrated the multifaceted constitution of child mortality in Kenya. The clear departure from the pre-2003 U5MR trends underscores the importance of macro-level factors in the improvement of intervention coverage and reduction of preventable child deaths. Kenya has already taken important steps to help reach SDG targets for child health, including its renewed commitment to achieving equity in access to health services. Kenya should continue to support its devolved structures through targeted policies and evidence-based programming, a bolstered health workforce and the sustained abolishment of user fees. To further the decline in U5MR, we have shown that the GoK must invest in maternal education, sexual and reproductive health and the nutrition of mothers and infants, including promotion of early breast feeding. Kenya must be prepared to strengthen national support for women’s reproductive health rights, especially given the recent US cuts to foreign aid for organisations providing counselling, referrals or services relating to abortion. Lastly, there is an urgent need to address the proliferation of urban settlements in Kenya, along with the vulnerable populations that reside within. In conclusion, Kenya is on the right track. With strengthened support for RMNCAH, including addressing components of the health system, and a dedicated focus in priority areas, Kenya’s SDG targets for children will be easily attainable.
