# Supplementary material for: Accelerating Kenya’s progress to 2030: understanding the determinants of under-five mortality from 1990 to 2015
Source: BMJ Glob Health. 2018 May 24;3(3):e000655. doi: 10.1136/bmjgh-2017-000655 (PMC5969726; doi:10.1136/bmjgh-2017-000655)

Accelerating Kenya's Progress to 2030: Understanding the Determinants of Under-Five Mortality from 1990 to 2015

Supplementary Material

TABLES

eTable 1: Interventions included in the composite coverage index (CCI).

Indicator		Definition
**CCI**	Composite coverage index	CCI is calculated as the weighted average of coverage of a set of eight preventive and curative interventions; it gives equal weight to four stages in the continuum of care: family planning, maternal and newborn care, immunization, and case management of sick children. The following expression is used to obtain the estimate: where FPS is demand for family planning satisfied, SBA is skilled birth attendant, ANCS is antenatal care with skilled provider, DPT3 is three doses of DPT vaccine, MSL is measles vaccination, BCG is BCG vaccination, ORT is oral rehydration therapy and continued feeding for children with diarrhoea, and CPNM is care seeking for children with suspected pneumonia. $CCI = {}^{1}/_{4}\left(FPS + \frac{SBA+ANCS}{2} + \frac{2DPT3+MSL+BCG}{4} + \frac{ORT+CPNM}{2}\right)$

eTable 2: Correlation matrix for predictor variables (2014).

	Maternal education	Paternal education	Wealth index	Region	Urban/ rural	Improved water	Improved sanitation	Maternal age at first birth	Number of children	CP
**Maternal education**	1									
**Paternal education**	0.6650*	1								
**Wealth index**	0.6050*	0.5817*	1							
**Region**	-0.2223*	-0.1850*	-0.1792*	1						
**Urban/ rural**	0.2390*	0.2529*	0.4129*	-0.1061*	1					
**Improved water**	0.2244*	0.2100*	0.3693*	-0.0427*	0.2218*	1				
**Improved sanitation**	0.2225*	0.2275*	0.3206*	-0.0673*	0.1072*	0.1072*	1			
**Maternal age at first birth**	0.1540*	0.0839*	0.1219*	-0.0282	0.0311	0.0634*	0.0614*	1		
**Number of children**	0.2919*	0.2011*	0.2310*	-0.1407*	0.1687*	0.1166*	0.0460*	0.0998*	1	
**CP**	-0.4286*	-0.3429*	-0.3792*	0.1574*	-0.1336*	-0.1231*	-0.0735*	-0.0529*	-0.1555*	1
**ANC**	0.2705*	0.2599*	0.2619*	-0.1312*	0.1727*	0.1431*	0.1253*	0.0896*	0.1514*	-0.1995*
**TT**	-0.0002	0.0065	-0.0059	-0.0341	0.0245	0.0232	0.0264	0.0027	0.0808*	0.0343
**SBA**	0.4228*	0.3740*	0.4653*	-0.1759*	0.2883*	0.1738*	0.1565*	0.0657*	0.2349*	-0.2680*
**Early BF**	-0.0283	-0.0194	-0.0109	0.0017	-0.0068	0.0087	0.0128	0.0178	-0.003	0.0219
**Birth order**	0.2882*	0.1927*	0.2290*	-0.1441*	0.1727*	0.0974*	0.0584*	0.0997*	0.8464*	-0.1485*
**Birth interval**	0.1450*	0.1405*	0.1533*	-0.1178*	0.0492*	0.0629*	0.0414*	-0.0078	0.0607*	-0.1208*

**Size at birth**	0.0698*	0.0345	0.0256	0.0399*	-0.0046	-0.0086	0.0171	0.0007	0.0328	-0.0403*
**Gender of infant**	0.0108	0.0289	0.0375	0.012	0.0244	0.008	0.0274	0.022	0.0216	-0.0229
**Maternal BMI**	0.0114	0.0157	-0.0612*	0.0269	-0.0739*	-0.0406*	-0.0587*	-0.0487*	-0.0473*	-0.0203
**Maternal weight**	0.3360*	0.3490*	0.4526*	-0.0929*	0.2239*	0.1903*	0.1775*	0.0811*	0.0333	-0.1837*
**Maternal height**	0.033	0.0480*	0.0571*	0.1596*	0.0386*	0.0513*	-0.0046	0.0333	-0.0122	0.0257

	**ANC**	**TT**	**SBA**	**Early BF**	**Birth order**	**Birth interval**	**Size at birth**	**Gender of infant**	**Maternal BMI**	**Maternal weight**
**Maternal education**										
**Paternal education**										
**Wealth index**										
**Region**										
**Urban/ rural**										
**Improved water**										
**Improved sanitation**										
**Maternal age at first birth**										
**Number of children**										
**CP**										
**ANC**	1									
**TT**	0.1010*	1								
**SBA**	0.3032*	0.0396*	1							
**Early BF**	0.0087	-0.0036	0.0281	1						
**Birth order**	0.1502*	0.0775*	0.2159*	-0.0314	1					
**Birth interval**	0.0712*	-0.0051	0.1357*	0.0127	0.0309	1				
**Size at birth**	0.0264	0.0156	0.0483*	0.0668*	0.0309	-0.004	1			
**Gender of infant**	0.0207	0.0094	0.0354	-0.005	0.0117	-0.0186	-0.0037	1		
**Maternal BMI**	-0.0233	-0.0391*	-0.0388*	-0.0358	-0.0348	-0.0474*	-0.0132	0.0123	1	
**Maternal weight**	0.1608*	0.0054	0.2713*	-0.0365	0.0401*	0.1122*	0.0540*	0.0417*	-0.0817*	1
**Maternal height**	-0.0119	-0.0093	-0.0083	0.0117	-0.015	-0.0124	0.0088	0.0052	-0.0346	0.3467*

CP=contraceptive prevalence; ANC=antenatal care; TT=tetanus toxoid vaccination; SBA=skilled birth attendant; BF=breastfeeding; BMI=body mass index.

eTable 3: Unadjusted and adjusted determinants of U5MR in Kenya, DHS 1993.

	Unadjusted			Adjusted		
Indicators	OR	95% CI	p-value	OR	95% CI	p-value
	Level 2: Intermediate level factors[a]					
**Maternal education (Ref: Secondary and above)**						
No formal education	1.15	0.53-2.51	0.73	--	--	--
Incomplete primary	1.44	0.67-3.06	0.35	--	--	--
Primary	0.77	0.36-1.65	0.50	--	--	--

**Paternal education (Ref: Secondary and above)**						
No formal education	1.76	1.06-2.90	0.03	1.84	1.12-3.03	0.016
Incomplete primary	1.41	0.91-2.20	0.13	1.38	0.89-2.14	0.153
Primary	1.06	0.68-1.64	0.80	1.07	0.69-1.64	0.77
**Wealth index (Ref: Richest)**						
Poorest	1.69	1.20-2.37	0.00	--	--	--
Poorer	1.38	0.98-1.95	0.07	--	--	--
Middle	0.93	0.64-1.34	0.69	--	--	--
Richer	0.89	0.61-1.31	0.56	--	--	--
**Region (Ref: Nairobi)**						
Central	0.51	0.25-1.05	0.069	0.45	0.07-0.45	0.069
Coast	1.00	0.52-1.92	0.995	0.80	1.00-0.80	0.553
Eastern	0.90	0.47-1.73	0.755	0.68	0.76-0.68	0.352
Nyanza	2.54	1.38-4.69	0.003	1.95	0.00-1.95	0.085
Rift Valley	0.58	0.31-1.10	0.094	0.42	0.09-0.42	0.032
Western	1.38	0.73-2.61	0.319	1.12	0.32-1.12	0.779
**Type of place of residence (Ref: Urban)****						
Rural	1.12	0.76-1.64	0.564	1.04	0.56-1.04	0.856
	Level 1: Proximal level factors[b]					
Household Characteristics:						
**Improved drinking water source (Ref: Yes)**						
No	1.04	0.83-1.30	0.761	--	--	--
**Improved sanitation facility (Ref: Yes)**						
No	1.69	1.15-2.49	0.007	--	--	--
Maternal Characteristics:						
**Maternal BMI (Ref: Normal 18.5-24.9)**						
Underweight: <18.5	0.96	0.66-1.40	0.85	1.04	0.63-1.72	0.89
Overweight: 25-29.9	1.03	0.73-1.46	0.86	0.92	0.54-1.59	0.775
Obese: >=30	2.44	1.77-3.38	<0.0001	2.51	1.54-4.10	<0.0001
**Maternal weight**	1.00	0.98-1.01	0.50	--	--	--
**Maternal height**	1.00	0.98-1.01	0.70	--	--	--
**Mother age at first birth (Ref: 25+)**						
<=15	1.08	0.59-1.97	0.815	--	--	--
16-25	0.77	0.44-1.37	0.376	--	--	--
**Fertility (Ref: 1-2 child)**						
3-4	0.99	0.76-1.30	0.966	1.85	1.02-3.34	0.043
>=5	1.27	1.00-1.61	0.053	1.36	0.74-2.51	0.322
Maternal Interventions:						
**Contraceptive Use (Ref: Yes)**						
No	1.82	1.41-2.34	<0.0001	0.75	0.52-1.07	0.11

**ANC visits (Ref: 4+ visits)**						
No ANC	1.97	1.24-3.14	<0.0001	1.33	0.73-2.42	0.346
<4 visits	1.34	1.05-1.71	0.02	1.26	0.94-1.70	0.123
**Not received 2 doses of TT**	0.99	0.76-1.28	0.95	--	--	--
**No SBA at birth**	1.24	1.00-1.54	0.05	--	--	--
**Early initiation of breastfeeding (Ref: <1 hour)**						
> 1 hour	2.88	2.25-3.68	<0.0001	2.37	1.68-3.34	<0.0001
Child Characteristics:						
**Birth order (Ref: 1-2)**						
3	1.06	0.85-1.31	0.619	--	--	--
4+	0.91	0.66-1.25	0.555	--	--	--
**Birth interval (Ref: 36+month)**						
<24 month	1.43	1.09-1.89	0.011	1.38	0.98-1.96	0.065
24-35 month	1.11	0.84-1.47	0.462	0.90	0.63-1.29	0.573
**Gender (Ref: Male)**						
Female	0.99	0.82-1.21	0.938	--	--	--
**Size at Birth (Ref: Average)**						
Small/very small	1.51	1.15-1.98	0.003	--	--	--
Large/very large	1.07	0.85-1.36	0.555	--	--	--

**Retained only at intermediate level
a-multivariable model with all intermediate level variables significant at p<0.20 in bivariate analysis and retained if significant at p<0.15, except area of residence
b- multivariable model with all proximal level variables significant in bivariate analysis at p<0.20 and all significant (p<0.15) intermediate level variables

eTable 4: Unadjusted and adjusted determinants of U5MR in Kenya, DHS 2003.

	Unadjusted			Adjusted		
Indicators	OR	95% CI	p-value	OR	95% CI	p-value
	Level 2: Intermediate level factors[a]					
**Maternal education (Ref: Secondary and above)**						
No formal education	2.42	1.59-3.68	<0.0001	1.86	1.08-3.21	0.025
Incomplete primary	2.29	1.56-3.37	<0.0001	1.93	1.22-3.05	0.005
Primary	1.67	1.13-2.46	0.01	1.54	1.00-2.37	0.053
**Father education (Ref: Secondary and above)**						
No formal education	2.06	1.46-2.90	<0.0001	2.04	1.31-3.16	0.002
Incomplete primary	1.79	1.31-2.43	<0.0001	1.57	1.11-2.22	0.011
Primary	1.37	1.04-1.81	0.027	1.25	0.92-1.69	0.154
**Wealth index (Ref: Richest)**						
Poorest	1.29	0.95-1.76	0.099	0.67	0.42-1.07	0.095
Poorer	1.06	0.76-1.48	0.724	0.60	0.38-0.97	0.036
Middle	1.03	0.74-1.43	0.88	0.71	0.45-1.13	0.148

Richer	0.91	0.64-1.29	0.597	0.74	0.48-1.15	0.183
**Region (Ref: Nairobi)**						
Central	0.61	0.36-1.03	0.066	0.62	0.33-1.17	0.142
Coast	1.02	0.62-1.67	0.947	0.91	0.52-1.60	0.747
Eastern	0.91	0.55-1.49	0.696	0.85	0.46-1.57	0.596
Nyanza	2.23	1.43-3.47	0	2.31	1.35-3.97	0.002
Rift Valley	0.93	0.59-1.46	0.743	0.73	0.42-1.27	0.27
Western	1.60	1.02-2.52	0.042	1.61	0.93-2.80	0.089
North Eastern	1.57	0.93-2.64	0.089	0.98	0.51-1.91	0.963
**Type of place of residence (Ref: Urban)****						
Rural	1.08	0.83-1.41	0.551	1.03	0.70-1.53	0.871
	Level 1: Proximal level factors[b]					
Household Characteristics:						
**Improved drinking water source (Ref: Yes)**						
No	1.26	1.01-1.57	0.041	--	--	--
**Improved sanitation facility (Ref: Yes)**						
No	1.15	0.86-1.55	0.353	--	--	--
Maternal Characteristics:						
**Maternal BMI (Ref: Normal 18.5-24.9)**						
Underweight: <18.5	1.05	0.77-1.43	0.76	0.64	0.33-1.24	0.184
Overweight: 25-29.9	1.08	0.80-1.46	0.61	1.10	0.63-1.91	0.736
Obese: >=30	1.40	1.02-1.91	0.04	1.98	1.19-3.30	0.009
**Maternal weight**	1.00	1.00-1.01	0.31	--	--	--
**Maternal height**	1.00	0.98-1.01	0.90	--	--	--
**Mother age at first birth (Ref: 25+)**						
<=15	1.52	0.87-2.67	0.144	--	--	--
16-25	1.08	0.65-1.79	0.777	--	--	--
**Fertility (Ref: 1-2 child)**						
3-4	1.65	1.29-2.11	<0.0001	--	--	--
>=5	1.88	1.48-2.40	<0.0001	--	--	--
Maternal Interventions:						
**Contraceptive use (Ref: Yes)**						
No	1.96	1.55-2.50	<0.0001	0.65	0.41-1.02	0.06
**ANC visits (Ref: 4+ visits)**						
No ANC	1.73	1.08-2.76	0.02	1.46	0.80-2.65	0.213
<4 visits	1.71	1.20-2.44	0.00	1.52	1.01-2.30	0.045
**Not received 2 doses of TT**	2.60	1.75-3.85	<0.0001	--	--	--
**No SBA at birth**	1.06	0.86-1.30	0.59	--	--	--
**Early initiation of breastfeeding (Ref: <1 hour)**						
> 1 hour	2.03	1.67-2.48	<0.0001	2.03	1.40-2.94	<0.0001

Child Characteristics:						
**Birth order (Ref: 1-2)**						
3	1.26	1.03-1.56	0.028	1.60	1.05-2.44	0.03
4+	1.15	0.87-1.51	0.338	1.41	0.82-2.40	0.211
**Birth interval (Ref: 36+ month)**						
<24 month	1.58	1.22-2.03	<0.0001	--	--	--
24-35 month	0.88	0.67-1.15	0.345	--	--	--
**Gender (Ref: Male)**						
Female	0.74	0.62-0.90	0.002	0.60	0.42-0.85	0.005
**Size at birth (Ref: Average)**						
Small/very small	1.81	1.43-2.31	<0.0001	1.50	0.95-2.36	0.084
Large/very large	1.18	0.93-1.49	0.172	1.19	0.78-1.80	0.419

**Retained only at intermediate level
a-multivariable model with all intermediate level variables significant at p<0.20 in bivariate analysis and retained if significant at p<0.15, except area of residence
b- multivariable model with all proximal level variables significant in bivariate analysis at p<0.20 and all significant (p<0.15) intermediate level variables

eTable 5: Unadjusted and adjusted determinants of U5MR in Kenya, DHS 2014.

	Unadjusted			Adjusted		
Indicators	OR	95% CI	p-value	OR	95% CI	p-value
	**Level 2: Intermediate level factors[a]**					
**Maternal education** (Ref: Secondary and above)						
No formal education	1.13	0.89-1.45	0.309	1.40	1.04-1.90	0.028
Incomplete primary	1.40	1.13-1.75	0.003	1.41	1.10-1.80	0.006
Primary	1.17	0.94-1.46	0.163	1.13	0.90-1.43	0.291
**Paternal education** (Ref: Secondary and above)						
No formal education	1.00	0.72-1.39	0.994	--	--	--
Incomplete primary	1.21	0.90-1.64	0.208	--	--	--
Primary	1.07	0.82-1.40	0.613	--	--	--
**Wealth index** (Ref: Richest)						
Poorest	1.14	0.90-1.46	0.273	1.23	0.90-1.68	0.191
Poorer	1.28	0.99-1.65	0.057	1.32	0.97-1.79	0.073
Middle	1.34	1.03-1.74	0.027	1.43	1.06-1.93	0.018
Richer	1.19	0.91-1.56	0.215	1.25	0.94-1.66	0.121
**Region** (Ref: Nairobi)						
Central	0.69	0.44-1.08	0.104	0.65	0.41-1.04	0.07
Coast	0.68	0.45-1.03	0.071	0.58	0.37-0.90	0.014
Eastern	0.55	0.36-0.83	0.005	0.47	0.30-0.73	0.001
Nyanza	0.88	0.59-1.32	0.551	0.76	0.50-1.17	0.209
Rift Valley	0.51	0.35-0.76	0.001	0.44	0.29-0.67	0
Western	0.68	0.44-1.05	0.079	0.57	0.36-0.90	0.016
North Eastern	0.53	0.33-0.84	0.007	0.41	0.25-0.69	0.001
**Type of place of residence** (Ref: Urban)						
Rural	0.93	0.80-1.09	0.378	0.87	0.73-1.03	0.113

	Level 1: Proximal level factors[b]					
Household Characteristics:						
**Improved drinking water source** (Ref: Yes)						
No	1.01	0.88-1.17	0.854	--	--	--
**Improved sanitation facility** (Ref: Yes)						
No	1.31	1.08-1.59	0.006	1.53	0.91-2.56	0.109
Maternal Characteristics:						
**Maternal BMI** (Ref: Normal 18.5-24.9)						
Underweight: <18.5	0.91	0.65-1.27	0.57	--	--	--
Overweight: 25-29.9	1.01	0.78-1.32	0.91	--	--	--
Obese: >=30	1.03	0.88-1.20	0.74	--	--	--
**Maternal weight**	1.00	1.00-1.01	0.28	--	--	--
**Maternal height**	0.99	0.97-1.00	0.15	--	--	--
**Mother age at first birth** (Ref: 25+)						
<=15	1.11	0.78-1.58	0.568	--	--	--
16-25	1.02	0.75-1.38	0.896	--	--	--
**Fertility** (Ref: 1-2 child)						
3-4	1.64	1.37-1.96	<0.0001	2.32	1.48-3.65	0
>=5	1.99	1.67-2.37	<0.0001	1.49	0.94-2.36	0.092
Maternal Interventions:						
**Contraceptive Use** (Ref: Yes)						
No	1.53	1.24-1.89	<0.0001	0.54	0.37-0.79	0.001
**ANC visits** (Ref: 4+ visits)						
No ANC	2.19	1.56-3.08	<0.0001	2.62	1.41-4.86	0.002
<4 visits	0.99	0.77-1.27	0.92	1.04	0.70-1.54	0.838
**Not received 2 doses of TT**	1.18	0.73-1.89	0.50	--	--	--
**No SBA at birth**	1.08	0.93-1.24	0.32	--	--	--
**Early initiation of breastfeeding** (Ref: <1 hour)						
> 1 hour	3.24	2.59-4.06	<0.0001	2.99	2.10-4.26	0.000
Child Characteristics:						
**Birth order** (Ref: 1-2)						
3	1.21	1.05-1.41	0.011	--	--	--
4+	0.99	0.81-1.22	0.936	--	--	--
**Birth interval** (Ref: 36+ months)						
<24 month	1.69	1.41-2.03	<0.0001	--	--	--
24-35 month	0.99	0.81-1.20	0.899	--	--	--
**Gender** (Ref: Male)						
Female	0.85	0.74-0.97	0.019	--	--	--
**Size at Birth** (Ref: Average)						
Small/very small	2.07	1.62-2.66	<0.0001	1.51	0.97-2.35	0.069
Large/very large	1.29	1.00-1.66	0.051	1.16	0.76-1.76	0.498

**Retained only at intermediate level
a-multivariable model with all intermediate level variables significant at p<0.20 in bivariate analysis and retained if significant p<0.15.
b-multivariable model with all proximal level variables significant in bivariate analysis at p<0.20 + significant (p<0.15) intermediate level variables.

FIGURES

eFigure 1: Conceptual framework for the Kenya Countdown case study.

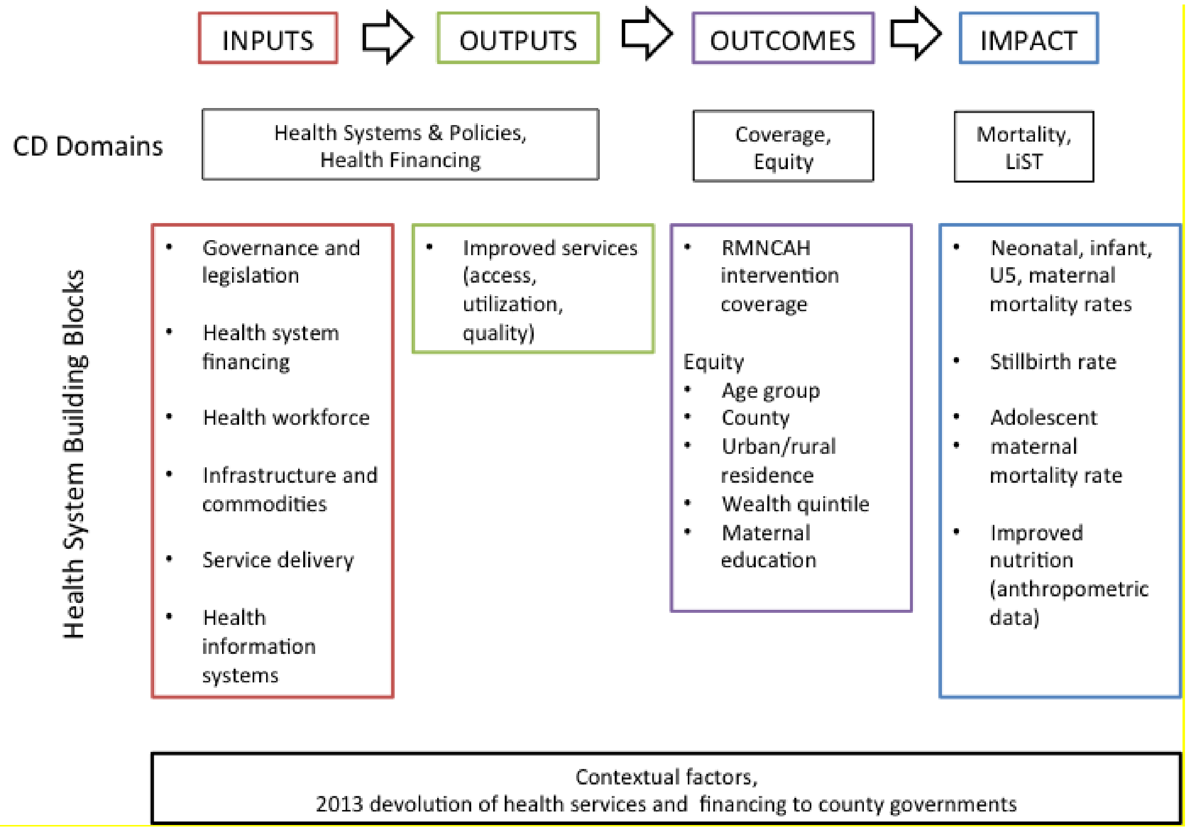

eFigure 2: Available Integrated Community Case Management (iCCM) policies for reducing under-five mortality (2014).

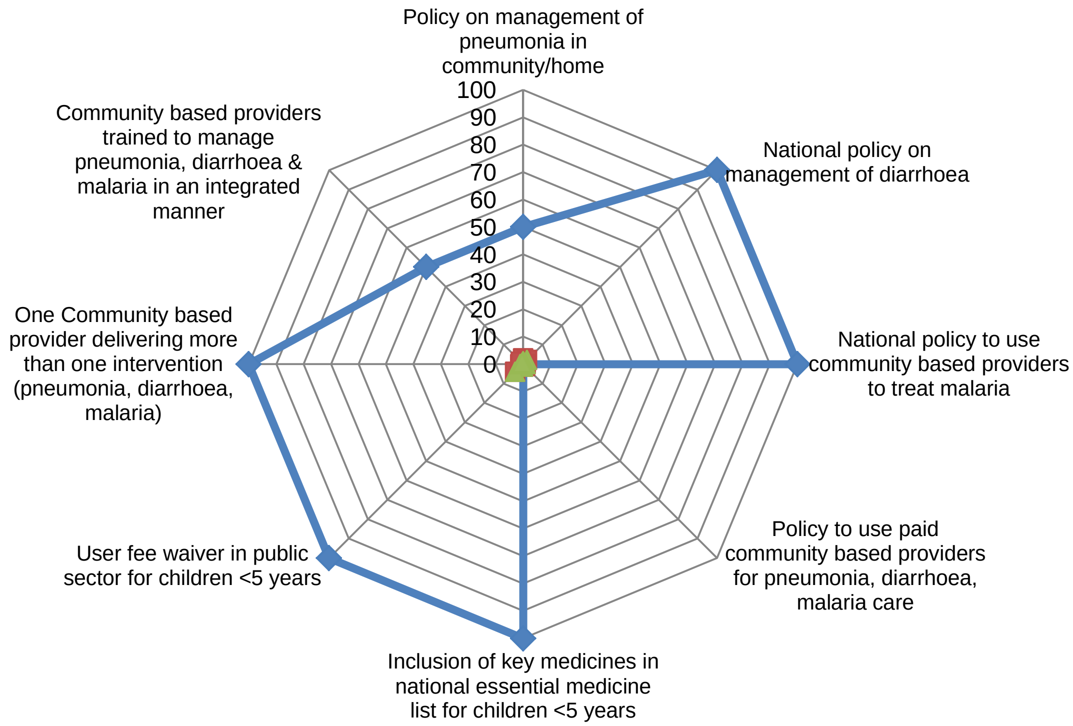

eFigure 3: Health facility availability of life saving commodities and composite coverage index (2014).

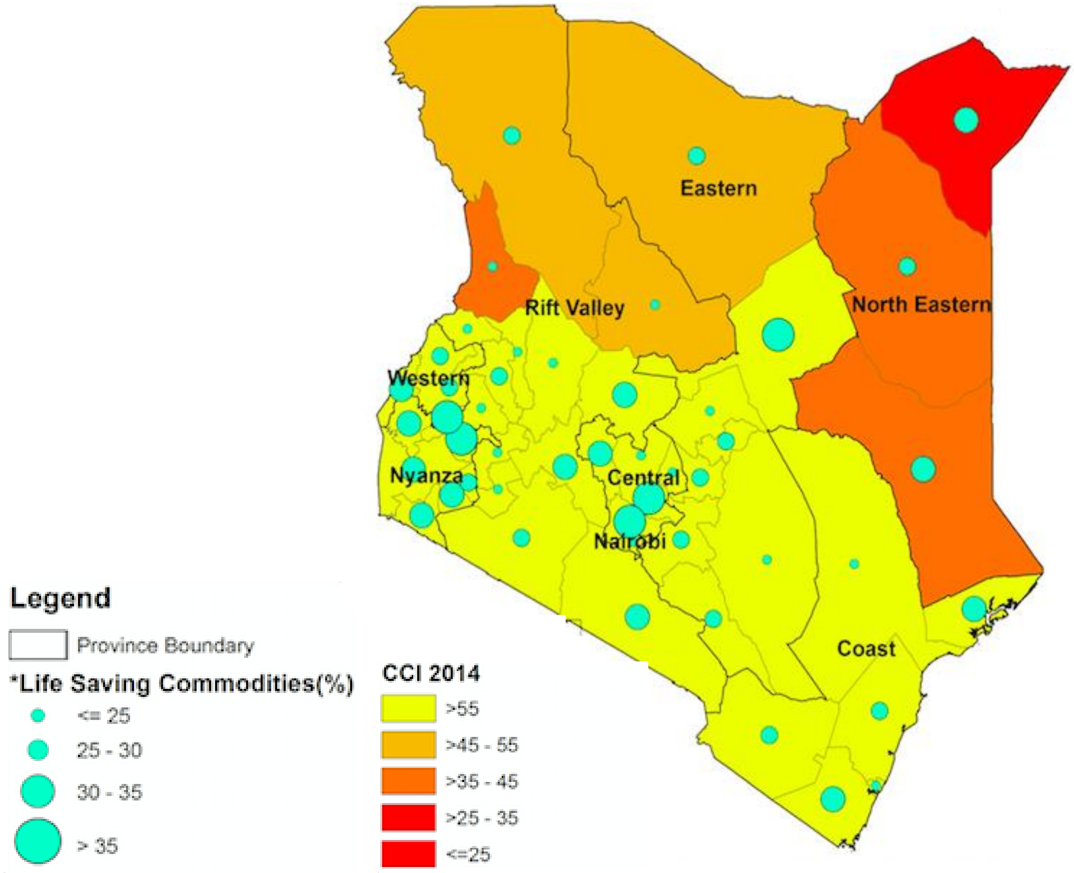

Life saving commodities: i) maternal = oxytocin, misoprostol, magnesium sulphate, ii) newborn = procaine benzylpenicillin, gentamicin, ceftriaxone, antenatal corticosteroids, skin disinfectant, iii) child = amoxicillin syrup, amoxicillin 500mg cap/tab, oral rehydration salts, zinc, and iv) reproductive health = female condoms.

eFigure 4: Health spending trends in Kenya between 2000 and 2013.
a. Total health spending (KES) and spending per capita (USD).

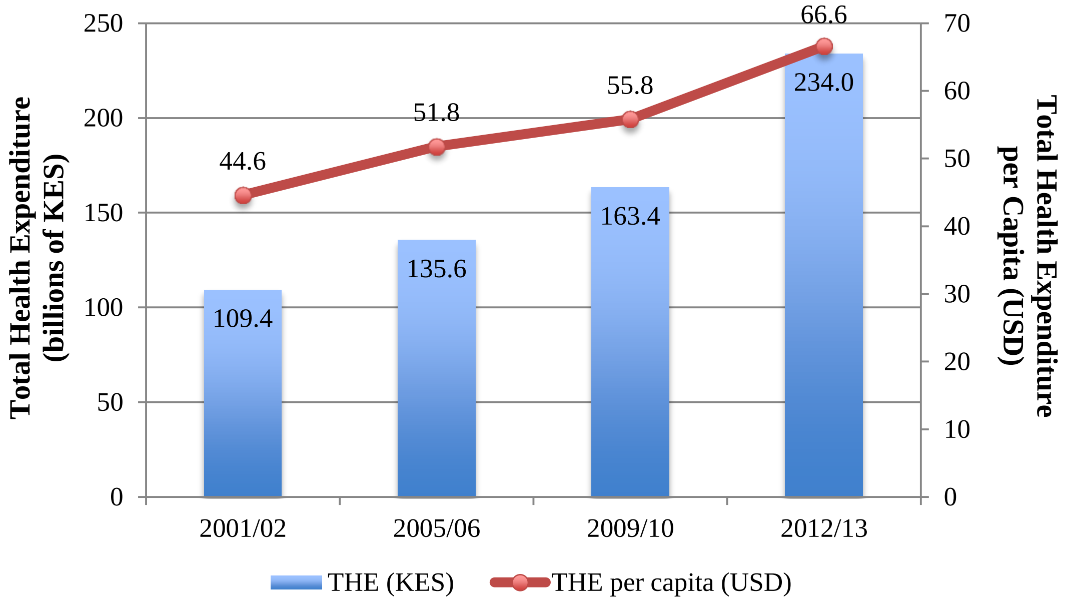

b. Health spending as a share of total government spending.

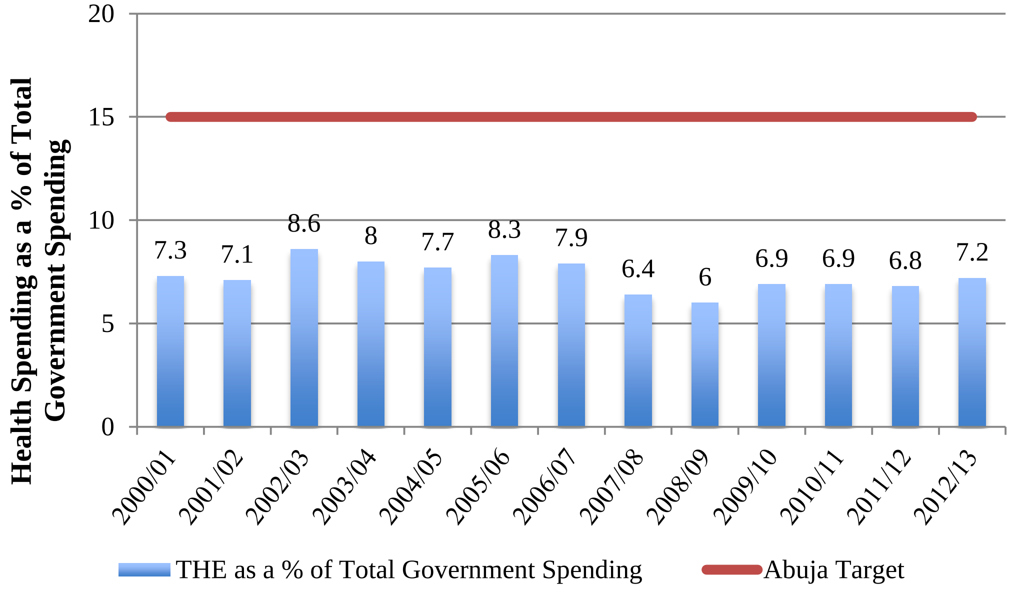

eFigure 5: HIV/AIDS epidemic trends in Kenya from 1990 to 2015.
a. National and sub-national HIV prevalence estimates among adults (15-49 years).

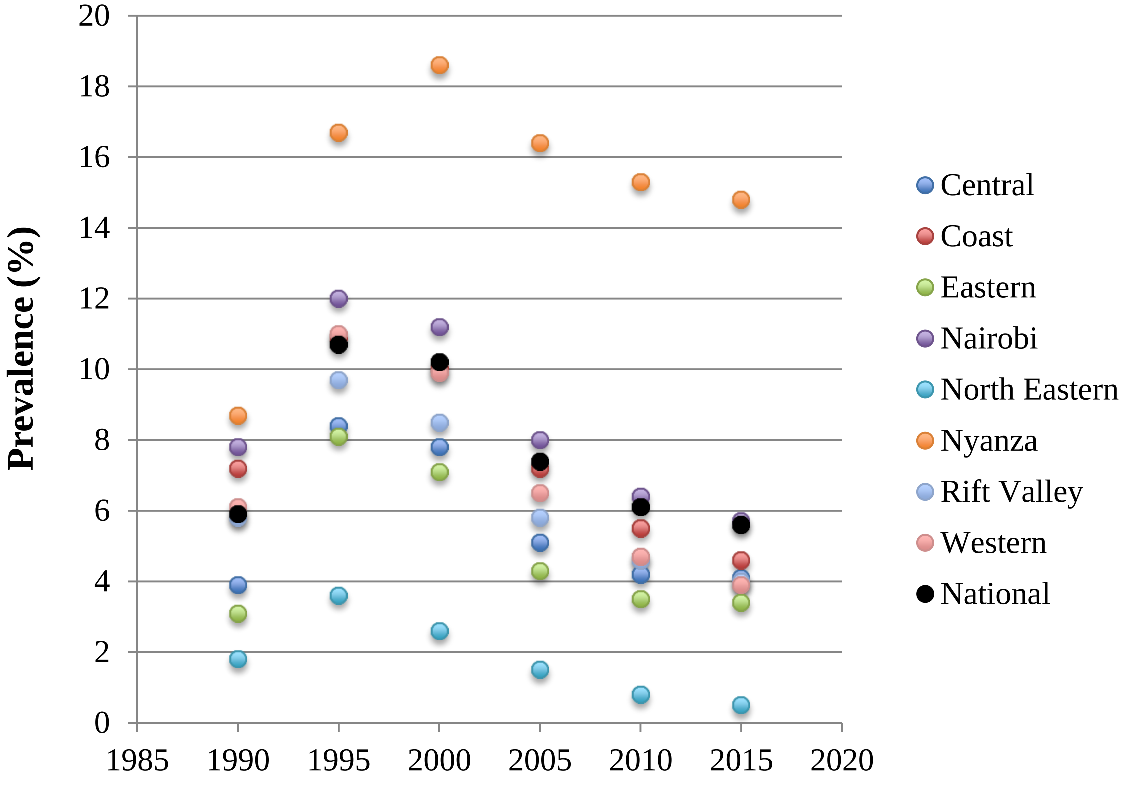

b. Trends in AIDS-related deaths among children (0-14 years) and child mortality (under-five deaths/1000 live births).

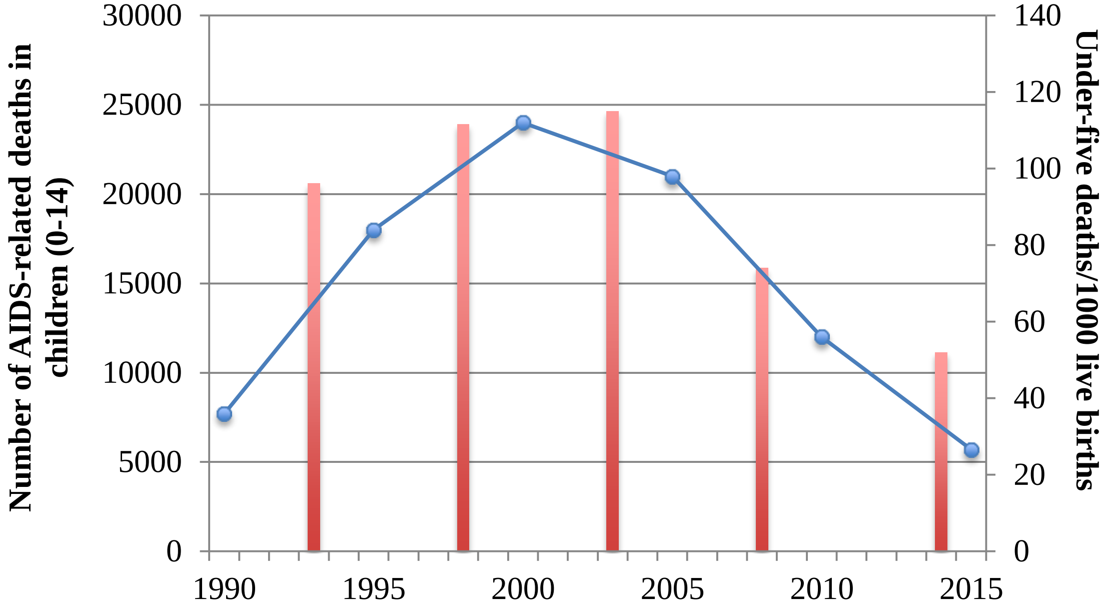

Supplement: Supplementary file 1 [file bmjgh-2017-000655supp001.pdf]
